# The True Cost of Heat: Evaluating Heat‐Related Mortality Estimation Methods in Texas

**DOI:** 10.1029/2025GH001537

**Published:** 2026-02-11

**Authors:** Jesse R. J. Rutt, Andrew E. Dessler

**Affiliations:** ^1^ Texas A&M University College Station TX USA

**Keywords:** heat‐related mortality, climate change, Texas, excess deaths, extreme heat, public health

## Abstract

Different ways of counting heat‐related deaths (HRD) can give you very different numbers. This study examines HRD in Texas using three different definitions: The Optimal Temperature Method (OTM) estimates mortality based on deviations from a community's optimal temperature, capturing the effects of both moderate and extreme heat exposure. This method finds that 2.2% of summertime Texas mortality were heat related over the period 2010–2023. The Extreme Heat Method (XHM) counts deaths associated with extreme temperatures; we find that temperatures exceeding the 95th percentile were responsible for 0.5% of summertime Texas mortality over this same period. This means that moderate heat is responsible for 77% of HRD, with extreme temperatures responsible for the rest. The Excess Death Method (EDM) approach quantifies the mortality burden as the increase in mortality compared to what would have occurred with the climate of a baseline period; we find that summertime Texas mortality over this period was 1.7% higher than with the climate of the mid‐20th century. When comparing these estimates to official HRD values from the State of Texas, which are based on an official determination that heat contributed to the death, we find that official State HRD numbers appear to substantially underreport HRD, attributing just 0.3% of summertime deaths to heat, with the XHM method being the closest.

## Plain Language Summary

This analysis reveals that heat kills far more people in Texas than official records show, with the death toll varying dramatically depending on how you count heat‐related deaths. Using three different methods to analyze 2010–2023 data, researchers found that the Optimal Temperature Method (counting all deaths above ideal temperature) estimated 2.2% of summer deaths were heat‐related, while the Extreme Heat Method (focusing only on the hottest days above 95th percentile) found 0.5% were from extreme heat, and the Excess Death Method (comparing to 1950s–60s baseline) attributed 1.7% to climate change. This shows that most heat deaths occur during moderately hot weather rather than extreme heat waves, four times as many people die from regular hot days as from extreme temperatures. Official Texas records captured only about one‐sixth of the statistically estimated heat deaths, though this undercounting has improved over time. These findings highlight the need for better heat death tracking systems and expanded protection programs for both extreme heat waves and routine hot weather as climate change brings more frequent and intense heat to Texas.

## Key Points

- Estimates of heat‐related mortality vary by a factor of four depending on the estimation method applied
- During extreme heat years 6% of summer deaths in Texas are heat related, with 2% caused by extreme heat
- Official records underestimate heat‐related deaths in Texas by a factor of six

## 1 Introduction

As global temperatures rise, heat‐related mortality is becoming an increasingly important public health concern (Ebi et al., 2021; Lüthi et al., 2023). High ambient temperatures can overwhelm the body's ability to regulate internal temperature, exacerbating pre‐existing conditions and increasing the risk of mortality, particularly among vulnerable populations such as the elderly, outdoor workers, and economically disadvantaged communities (Hajat et al., 2010). However, quantifying heat‐related deaths (HRD) remains a challenge.

To address this, researchers have developed multiple ways to count HRD, each offering distinct insights into how heat affects mortality but leading to widely varying estimates of HRD. Each approach has distinct advantages and is suited to answering specific research questions, whether related to public health planning, emergency response, or long‐term climate risk assessment.

The most common approach in the academic literature is the optimal temperature method (hereafter, OTM). This method counts heat‐related deaths occurring at temperatures above the optimal temperature (OT), at which the fewest deaths occur, which is also referred to as the minimum mortality temperature (MMT) (Gasparrini et al., 2015). This approach captures the effects of both moderate and extreme heat exposure, making it valuable for understanding long‐term heat‐related health impacts. For example, a recent study by Ballester et al. (2023) used this method to estimate that 61,672 heat‐related deaths occurred across Europe in the summer of 2022.

Another method of quantifying HRD is to count mortality during the hottest temperatures, typically defined by temperatures above the 90th or 95th percentile (Anderson & Bell, 2011; De'Donato et al., 2018), which we refer to as the Extreme Heat Method (XHM). This approach is particularly useful for emergency response planning and short‐term interventions. A study by Anderson and Bell (2011) applied a similar method to 43 U.S. cities and found that mortality risk increased by 3.7% during periods with temperatures above the 95th percentile. Given that the OT is lower than the 95th percentile temperature, the OTM will always give a higher count of HRD than the XHM.

The final method of quantifying HRD is the Excess Death Method (EDM), which estimates total HRD during a period of interest by comparing OTM estimates during the period to OTM estimates using the climate of a baseline period (Ballester et al., 2023; Ferenci, 2023; Guo et al., 2016; Robine et al., 2008). In other words, this provides an estimate of how many additional deaths occurred during a particular event compared to what you would expect from the baseline climate. For example, Robine et al. (2008) applied a similar method to estimate over 70,000 excess deaths across Europe during the 2003 heatwave compared to the baseline climate of 1998–2002, a figure that far exceeded early official estimates. The Ballester et al. (2023) study of the 2022 European heat wave also applied this approach, estimating that 25,561 excess deaths occurred in 2022 compared to a baseline from 2015 to 2021. This estimate, which is less than half the 61,000 deaths calculated using the OTM, highlights how different methodological choices can lead to substantially different estimates of HRD.

We estimate HRD using a single heat exposure framework and report the estimates using three complementary methods: OTM, XHM, and EDM. This study applies all three methods to analyze heat‐related mortality in Texas, a state that experiences frequent and severe heatwaves, significant urban heat island effects, and socioeconomic disparities that heighten vulnerability to extreme temperatures. By comparing these approaches to official State of Texas HRD numbers, we aim to understand the extent of HRD in Texas, evaluate how different methods influence mortality estimates, and determine which approaches are most appropriate for different public health and policy applications.

## 2 Methods

All calculations of HRD are based on the same relationship between temperature, mortality risk, and baseline mortality rate for each region. This section details the data sources, preprocessing steps, and methodological approaches used to estimate heat‐related deaths.

### 2.1 Data Sources and Preprocessing

#### 2.1.1 Temperature Data

Daily mean temperature data was obtained from the European Centre for Medium‐Range Weather Forecasts Reanalysis version 5 (ERA5) data set (Hersbach et al., 2020). ERA5 provides global data with 0.25° × 0.25° spatial resolution. The temperature assigned to each Texas county is from the ERA5 grid point nearest the county's centroid.

#### 2.1.2 Official Heat‐Related Death Data

Texas official HRD counts spanning 2010–2023 was provided by personal communication from Deceleration News, who requested it from the Texas Department of State Health Services via an open records request.

This data set contained annual counts of death certificates that either listed hyperthermia as an immediate cause of death or a contributing factor at the county totals. The Official counts for 2022 and 2023 were non‐final and thus there may be some changes to the official tallies in more recent data sets due to potential revisions in death certificate classifications and delayed reporting.

#### 2.1.3 Total Mortality Data

Total death counts were obtained from a data set spanning daily records for Texas counties from 2013 to 2022. This data set was provided by personal communication from The Texas Tribune and was obtained via an open records request of Texas Department of State Health Services.

### 2.2 Heat‐Related Death Modeling

In this study, we calculate heat‐related deaths (HRD) for June through September, which we refer to as summertime, when most heat‐related mortality occurs in Texas. We tested including additional months (e.g., May) but found that it changed the mortality estimates by less than 2%. To estimate heat‐related mortality, we first calculate a Relative Risk (RR) function, which quantifies the increase in mortality risk at temperatures exceeding the optimal temperature. This RR value is then used to estimate the number of HRD each day.

#### 2.2.1 Relative Risk

This study employs the Relative Risk (RR) parameterization from Shindell et al. (2020), who developed a generalized risk function for heat‐related mortality across the contiguous United States. Their research revealed that the temperature‐mortality relationship varies significantly by region, with cooler areas experiencing steeper mortality increases at high temperatures (see also (Lee & Dessler, 2023)).

To capture this spatial variation, Shindell et al. fit second‐order polynomials to risk curves from 10 climatically diverse U.S. cities. They then regressed these polynomial coefficients against Summer Mean Temperature (SMT) to create a general function. The resulting equation is:

(1) $$\text{RR}\left(T\right)=1+a_{s}\cdot \left(\text{SM}T_{c}-a_{i}\right)\cdot {\left(T-OT_{c}\right)}^{2}+b_{s}\cdot \left(\text{SM}T_{c}-b_{i}\right)\cdot \left(T-OT_{c}\right)$$

where *T* = daily mean temperature, O*T*_*c*_ = Optimal Temperature, which we assume is equal to the 84th percentile of daily mean temperatures from 2010 to 2023 for each county c (Gasparrini et al., 2015; Shindell et al., 2020), SMT_c_ = Summer Mean Temperature (the mean temperature from June to September, calculated over the period from 2010 to 2023) for county *c*. The constants *a*_*s*_ and *b*_*s*_ are slopes of coefficient fits, and *ai* and *bi* are intercepts of coefficient fits and are also taken from Shindell et al.

We calculate both the O*T*_*c*_ and SMT_c_ for each county using the same baseline period as our analysis window (2010–2023). This ensures that these values reflect the actual climate conditions experienced during the study period. To evaluate the sensitivity of the results to the use of different baseline definitions, we also repeated the calculation using the original 1985–2006 baseline that was used by Shindell et al. (2020). This yielded HRD estimates about 20% larger. This difference arises from the earlier baseline representing a cooler climate, which lowers the OT threshold and therefore classifies more days as heat exposed. Using a more recent baseline reduces HRD relative to historical baselines but may better capture population adaptation to current conditions (Barreca et al., 2016).

We understand that using SM*T*_*c*_ and O*T*_*c*_ from the modern period will tend to reduce the number of HRD relative to what we would calculate if we used SM*T*_*c*_ and O*T*_*c*_ from the period that the curves were derived from. But this seems reasonable given that we expect people will have better adapted to climate change since the 1985–2006 period (Barreca et al., 2016). Ultimately, this remains an uncertainty in our analysis.

#### 2.2.2 Calculation of Heat‐Related Deaths

Using Equation 1, we determine an R*R*_*c*_ curve for each county *c*. HR*D*_*c*_ for each county on a day with temperature *T* is then estimated as:

(2) $${\text{HRD}}_{c}=\left(\text{RR}{\left(T\right)}_{c}-1\right)\cdot \text{MMTD}\;{\left(y\right)}_{c}$$

where RR(*T*)_c_ is the relative risk for that county on a day with temperature *T* and MMTD(*y*)_c_ is the minimum mortality temperature death rate for county *c* during year *y*. Daily HRD values are first computed for each county and then summed to produce monthly and annual totals. State‐level HRD estimates are obtained by summing across all counties, representing the total estimated heat‐related deaths in Texas.

MMTD(*y*)_c_ is calculated separately for each county *c* and each year *y* by averaging total death counts on days where *T* falls within ±2°C of the OT. We calculate MMTD(*y*)_c_ values this way for individual years from 2013 to 2019 and linearly extrapolate for years before 2013 and after 2019. This extrapolation approach avoids the anomalously high mortality observed during the COVID‐19 pandemic years (after 2020) while still accounting for long‐term changes due to population growth, healthcare advancements, and climate adaptation.

In Section 3, we apply this common framework using three methods to produce heat‐related mortality estimates. Specifically, the Optimal Temperature Method (OTM) sums daily HRD across all days warmer than the OT, the Extreme Heat Method (XHM) restricts the summation to only the hottest days (≥95th percentile), and the Excess Death Method (EDM) compares OTM‐based estimates in the study period against those using the climate from the historical baseline. Thus, Section 3 presents different applications of the same underlying model described here, rather than distinct methodologies.

## 3 Results and Discussion

Across Texas counties, the optimal temperature (OT) ranges from 25.3°C to 30.2°C, with a statewide mean of 28.5°C. The minimum mortality temperature death rate (MMTD) which represents the daily baseline mortality at the optimal temperature varies significantly with population size, ranging from fewer than 1 death per day in rural counties to more than 80 deaths per day in the large urban counties. The calendar day of minimum mortality most often falls in late June. Relative risk values increase steadily above OT, with mortality risks reaching approximately 1.10–1.25 at the 95th percentile of daily mean temperature, depending on county. These RR magnitudes are consistent with prior U.S. centric studies (Guo et al., 2023; Shindell et al., 2020; Weinberger et al., 2020).

### 3.1 Heat‐Related Mortality Estimates by Method

#### 3.1.1 Optimal Temperature Method

**Figure 1:**
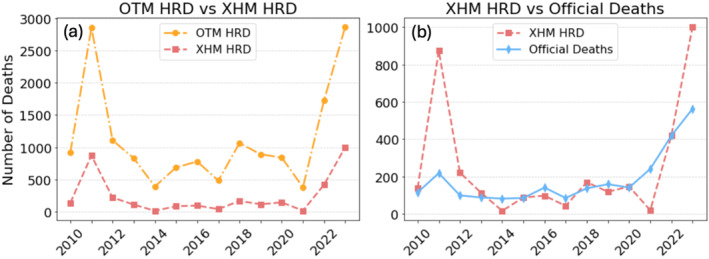
(a) Comparison of modeled HRD from the Optimal Temperature Method (OTM) and Extreme Heat Method (XHM) methods from 2010 to 2023. (b) Comparison of modeled HRD from XHM and officially reported HRD from the State of Texas.

The OTM counts all mortality occurring above the OT and it consistently produces the highest estimates of heat‐related mortality in Texas (Figure 1a). Over the 2010–2023 period, the OTM estimates a total of 15,826 summertime HRD in Texas, an average of 1,130 summertime deaths per year (Figure 1a). This means that 2.2% of all summer deaths were caused by moderate and extreme heat.

HRD estimates under the OTM fluctuate significantly from year to year, reflecting interannual variations in summer heat. The hottest years, 2011 and 2023, resulted in high mortality, with 2,854 and 2,867 deaths, respectively (6.3% and 4.8% of summer deaths respectively). Years with milder summer conditions, such as 2014 and 2021, had 393 and 381 deaths, representing just 0.9% and 0.6% of summer deaths, respectively, about one seventh of the HRD in the hottest years.

#### 3.1.2 Extreme Heat Method

The XHM counts mortality during extreme temperatures (in our case, above the 95th percentile), so it produces lower HRD estimates than the OTM (Figure 1). Over the 2010–2023 period, the XHM estimates a total of 3,470 summertime HRD in Texas, an average of 248 deaths per year. Extreme heat therefore accounts for 0.5% of all summer HRD across the study period.

XHM variability follows that of OTM (Figure 1a), with notable peaks in the hottest years. The hottest years, 2011 and 2023, resulted in high mortality, with 873 deaths and 1,002 deaths, respectively (1.9% and 1.7% of summer deaths respectively). In contrast, years with milder summer conditions, such as 2014 and 2021, had lower XHM estimates, 16 and 20 deaths, representing just ∼0.03% of summer deaths.

The difference between the OTM and XHM numbers is the contribution of moderate heat, meaning temperatures between the OT (the 84th percentile) and 95th percentiles. While extreme heat waves receive much of the attention in public health discussions, we infer from Figure 1 that the majority of HRD actually occurs during these moderate heat days (Gasparrini et al., 2015). For example, in 2011, moderate heat accounted for 4.4% of summer deaths, more than double the number of deaths attributed to extreme heat. Even in years without exceptional heat, moderate heat remains a persistent and substantial contributor to HRD.

#### 3.1.3 Excess Death Method

The EDM during period *x* is equal to:

(3) $${\text{EDM}}_{x}={\text{OTM}}_{x}-{\text{OTM}}_{B}$$

where EDM_*x*_ is the excess deaths during period *x*, OTM_*x*_ is the estimate of HRD from the OTM method during period *x*, and OTM_B_ is the OTM estimate of HRD using the climate of a baseline period. EDM therefore provides a direct measure of how climate change since the baseline contributed to HRD during period *x*. This method is particularly valuable for assessing the broader, systemic impact of climate change on HRD.

**Figure 2:**
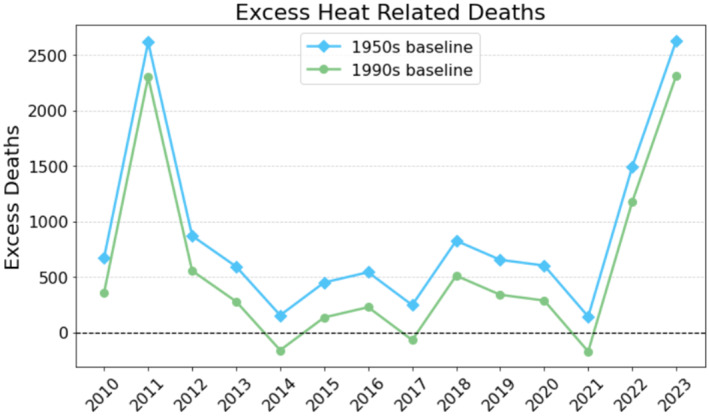
Annual excess HRD estimated using the OTM from 2010 to 2023, relative to two baseline periods: 1950–1963 (blue) and 1990–2003 (green).

Using baseline temperatures from the 1950–1963 period, the EDM results indicate a substantial increase in HRD over time. Over the 2010–2023 period, there were a total of 12,481 excess deaths compared to HRD from the climate of the historical baseline, an average of 891 additional summertime deaths per year (Figure 2). This means that climate change since the middle of the 20th century is causing 1.7% of all summer deaths in Texas.

The EDM shows similar interannual variability. In 2011 and 2023, the EDM estimates 2,615 and 2,300 excess deaths, respectively, representing 5.81% and 3.83% of all summer deaths (Figure 2). In contrast, years with milder summer conditions, such as 2014 and 2021, yielded far lower excess mortality. The EDM estimated 153 and 141 excess deaths in those years.

EDM estimates are always tied to a reference period. To test our sensitivity to the reference period, we have recalculated the EDM estimates using the 1990–2003 period for reference (Figure 2). Because the reference period is hotter, EDM predicts fewer excess deaths. For example, under this 1990–2003 baseline, the average annual excess mortality drops from 891 deaths per year to 576 deaths per year. In 2023 alone, the EDM estimate declines from 2,627 to 2,312 under the later baseline. Yearly values are shown in Table 1.

**Table 1:** Annual and Total Heat‐Related Death (HRD) Estimates in Texas From 2010 to 2023 Using the Optimal Temperature Method (OTM), Extreme Heat Method (XHM), Excess Death Method (EDM, Relative to 1950–1963 Baseline), and Official Death Certificate Counts

Year	OTM HRD	XHM HRD	EDM HRD	Official HRD
2010	915	138	675	115
2011	2,854	873	2,615	218
2012	1,108	222	869	99
2013	831	113	592	88
2014	393	16	153	82
2015	688	88	448	85
2016	780	98	541	141
2017	484	43	245	85
2018	1,064	170	825	137
2019	892	118	653	159
2020	840	147	601	141
2021	381	20	141	241
2022	1,729	422	1,489	419
2023	2,867	1,002	2,627	563
Total	15,826	3,470	12,481	2,573
Average	1,130	248	891	184

### 3.2 Comparison to Official Heat‐Related Death Records

#### 3.2.1 Optimal Temperature Method Versus Official Records

The OTM consistently yields the highest HRD estimates; the ratio of OTM to official deaths is 6.15 over the entire period (Figure 1 and Table 1).

The wide difference between OTM and official HRD counts likely stems from the difficulty of officially attributing deaths to heat when temperatures are moderate, when temperature is a contributing factor rather than a primary one. For example, a cardiovascular death on a moderately hot day may be triggered or accelerated by heat‐induced physiological stress, yet temperature is not the primary cause of death and may not be noted on the death certificate. We can only see the impact of moderate heat in population‐wide statistical modeling used in the OTM. However, the ratio declined from an average of 11 from 2010–2013 to 4.26 for 2020–2023. This decline in the difference over time suggests that the official estimates of mortality are improving.

#### 3.2.2 Extreme Heat Method Versus Official Records

The XHM yields more conservative HRD estimates compared to the OTM; the ratio of XHM to official deaths is 1.35 over the entire period (Figure 1 and Table 1). This ratio decreases from an average of 2.59 from 2010–2013 to 1.17 for 2020–2023.

The narrower difference between XHM and official counts compared to OTM likely stems from the fact that XHM focuses only on days with temperatures above the 95th percentile, where heat is a more important contributor to mortality. Consequently, the official attribution of these deaths to heat is more straightforward. Nevertheless, even with the XHM, undercounting may still occur due to challenges in identifying heat as a contributing factor even at these elevated temperatures. As with the OTM, the decline in the difference between XHM and official HRD estimates suggests that improvements in the official attribution of heat‐related deaths over time have led to better official estimates of mortality.

#### 3.2.3 Excess Death Method Versus Official Records

The EDM consistently yields higher HRD estimates compared to official records; the ratio of EDM to official deaths over the full period is 4.85, indicating that EDM estimates are substantially higher. As with the other metrics, the ratio decreases from an average of 9.14 from 2010–2013 to 3.56 for 2020–2023, likely reflecting improvements in identifying heat‐related mortality.

It's not surprising that the EDM estimates differ from the official HRD numbers since they are measuring different things. EDM measures the increase in HRD due to climate change, while the official numbers are intended to measure all heat‐related mortality.

## 4 Conclusions

This study provides a comparison of heat‐related death (HRD) metrics in Texas from 2010 to 2023, focusing on summer months (June–September). Our analysis demonstrates that different methodological approaches yield substantially different HRD estimates, with important implications for public health planning and climate adaptation strategies.

The Optimal Temperature Method (OTM), which counts all deaths occurring on days exceeding the optimal temperature (the temperature with the lowest mortality rate), consistently produces the highest estimates (2.2% of summertime deaths). The Extreme Heat Method (XHM), focusing only on mortality during the hottest days (above the 95th percentile), provides more conservative estimates (0.5% of summertime deaths), which agree more closely with official records (0.3% of summertime deaths). The Excess Death Method (EDM), which quantifies additional mortality attributable to climate change compared to a mid‐20th century baseline, indicates that 1.7% of summertime deaths can be linked to rising temperatures since the middle of the 20th century.

A key finding is that official death records from the State of Texas significantly underestimate heat‐related mortality. While the XHM shows the closest agreement with official counts during moderate years, it still identifies 35% more deaths than officially recorded over the full study period. This discrepancy highlights a critical gap in mortality surveillance, as death certificates often fail to attribute deaths to heat unless the deaths are very clearly related to heat. However, we also find that the disagreement between official HRD numbers and our statistical estimates is declining in time, with recent years showing less disagreement. One interpretation is that State officials are getting better at identifying heat‐related mortality.

The large difference between the OTM and XHM HRD estimates confirms that moderate heat exposure, temperatures above OT but below extreme thresholds, is responsible for the majority of heat‐related deaths (see also Gasparrini et al., 2015). This finding underscores the need for public health research to better understand how moderate heat exposure leads to mortality.

As Texas faces rising temperatures and more frequent extreme heat events due to climate change, these findings provide insights for developing targeted adaptation strategies. Improved mortality tracking systems, expanded cooling programs in vulnerable communities, and better risk communication for both extreme and moderate heat conditions are necessary to reduce the substantial and growing health burden of heat exposure.

## Conflict of Interest

The authors declare no conflicts of interest relevant to this study.

## Data Availability

The data and scripts used in this study are openly available: The all‐cause mortality and ERA5 temperature data as well as the Python scripts and data used for processing data, conducting analysis, and generating all figures in the study is preserved at Zenodo via https://doi.org/10.5281/zenodo.17188151 with a Creative Commons Attribution 4.0 International License (Rutt, 2025).
